# Volumetric Pulp Analysis of Mandibular Molars on CBCT as a Supplementary Indicator for Age-Related Dental Changes: A Preliminary Investigation in a Turkish Population Sample

**DOI:** 10.3390/diagnostics16162552

**Published:** 2026-08-13

**Authors:** Hatice Boyacioglu Erden, Nihal Avcu, Gokcen Akcicek, Nursel Akkaya, Sema Dural

**Affiliations:** Department of Dentomaxillofacial Radiology, Faculty of Dentistry, Hacettepe University, 06230 Ankara, Turkey; navcu@hacettepe.edu.tr (N.A.); gokcen.akcicek@hacettepe.edu.tr (G.A.); ynursel@hacettepe.edu.tr (N.A.); sdural@hacettepe.edu.tr (S.D.)

**Keywords:** cone-beam computed tomography, forensic age estimation, mandibular molar, pulp volume, robust regression, secondary dentine

## Abstract

**Background/Objectives:** This study aimed to evaluate the relationship between CBCT-derived total pulp volume and chronological age in mandibular first molars (M1) and second molars (M2), and to explore the preliminary utility of volumetric pulp analysis as a supplementary indicator for age-related dental changes in a Turkish population sample. **Methods:** CBCT scans of 60 individuals (32 females, 28 males; age range: 18–48 years) were retrospectively analysed. Pulp volumes of 60 mandibular molars (32 first and 28 second molars) were segmented using a semi-automatic thresholding approach with manual correction. Spearman correlation analysis was performed to assess the relationship between pulp volume and chronological age, stratified by sex and tooth type. Following comprehensive regression diagnostics—including the Shapiro–Wilk test, Breusch–Pagan test, and Cook’s distance—Huber M-estimator robust regression was selected as the primary analytical method, with OLS bootstrapped regression retained as a sensitivity analysis. **Results:** A statistically significant negative correlation was found between pulp volume and chronological age in both mandibular first molars (Spearman ρ = −0.554, *p* = 0.001) and second molars (ρ = −0.693, *p* < 0.001). Huber robust regression confirmed pulp volume as a significant predictor in both groups: M1 (β = −0.151, *p* = 0.001; Age = 33.646 − 0.151 × Volume) and M2 (β = −0.205, *p* < 0.001; Age = 36.833 − 0.205 × Volume). OLS sensitivity analysis yielded *R*^2^ = 0.238 (SEE = ±5.978 years, *n* = 32) for M1 and *R*^2^ = 0.499 (SEE = ±4.908 years, *n* = 28) for M2. No statistically significant sex differences were detected in either molar group. **Conclusions:** This preliminary study suggested that CBCT-derived mandibular molar pulp volumetry demonstrates a statistically significant negative association with chronological age in a predominantly young adult Turkish population.

## 1. Introduction

Secondary dentine deposition initiates after tooth eruption is completed, and the size of the pulp cavity decreases with age [1]. Gustafson first investigated this relationship for age estimation in 1950; his method was invasive, requiring tooth resection [2]. Subsequent non-invasive age estimation studies used photographs and dental radiological images for tooth measurements [3,4,5]. These measurements included one-dimensional linear measurements and two-dimensional area measurements. Kvaal’s linear measurement ratios showed high correlation with age, presenting determination coefficients ranging from 0.56 to 0.76 [6]. Cameriere’s pulp-tooth area ratio reported a higher determination coefficient value of 0.85 [7]. These methods were applied to other populations and found to be reproducible and applicable age estimation biomarkers in living individuals [8,9,10,11].

Radiological studies using panoramic and periapical images have notable drawbacks, including superimposition of structures, limited visibility of pulpal changes, and difficulty in identifying the overall shape of the tooth. The growth of cone beam computed tomography (CBCT) in dentistry has enabled researchers to assess pulp cavity size in three dimensions in living individuals, overcoming these limitations [12]. Microcomputed tomography (μCT) also offers the opportunity to evaluate pulp cavity volume; however, it is invasive and requires tooth extraction, precluding its use in living individuals. The correlation between age and secondary dentine deposition on μCT was first investigated by Vandevoort et al. in 2004 [13]. Further studies reported higher correlation coefficients. Someda et al. evaluated mandibular central incisors considering sex and reported the highest determination coefficient for the volume ratio of the pulp cavity to the whole tooth excluding the enamel [14]. Aboshi et al. assessed pulp–tooth volume ratios at different levels of mandibular premolar teeth with similar results [15]. Although CBCT scans of anterior and canine teeth are frequently chosen due to their simpler shape, volumetric data on secondary dentine accumulation in mandibular molars remain limited, and population-specific reference values for these teeth are correspondingly scarce. In addition, previous volumetric studies have commonly relied on ordinary least-squares regression without formally reporting whether its underlying assumptions were met, despite the sensitivity of such small-sample models to non-normal residuals and influential observations.

Therefore, the aim of this study was to investigate the relationship between CBCT-derived pulp volume and chronological age in mandibular first and second molars in a Turkish population sample, and to assess the potential utility of volumetric pulp analysis as a supplementary indicator within multi-factorial age estimation frameworks, using a statistically rigorous modelling strategy that explicitly accommodates potential regression assumption violations. Given the exploratory nature of the study and the inherent limitations of the available archival sample, the findings are intended to provide preliminary reference data and to identify directions for future methodologically refined investigations.

## 2. Materials and Methods

### 2.1. Ethical Approval and Study Design

This retrospective study was conducted in strict accordance with the Declaration of Helsinki and received formal approval from the Non-Interventional Ethics Committee of Hacettepe University (Protocol No: GO 15/803-25, Date: 16 December 2015). The approval covered the retrospective use of pre-existing, anonymised archival imaging data; all CBCT scans analysed had been acquired between 2014 and 2015 for routine clinical indications prior to the date of approval, and no imaging was performed for the purposes of this study. Written informed consent for the anonymised use of clinical records and imaging data in scientific research had been obtained from all patients at the time of their routine radiographic examination. All images were obtained using an i-CAT Next Generation scanner (Imaging Sciences International, Hatfield, PA, USA) with standardised acquisition parameters: 120 kVp, 5 mA, an exposure time of 14.7–17.8 s, a voxel size of 0.20 mm, and fields of view ranging from 16 × 8 cm to 16 × 13 cm.

### 2.2. Study Sample and Inclusion Criteria

The study sample consisted of 60 Turkish individuals (28 males and 32 females) aged between 18 and 48 years, with each patient contributing exactly one molar tooth (either M1 or M2) to avoid clustering of observations within individuals and the consequent violation of the independence assumption. Consequently, the first molar and second molar subgroups comprise mutually exclusive individuals, and all observations within each regression model are statistically independent. Inclusion criteria required the presence of the following teeth on at least one side: mandibular first molar (M1) or mandibular second molar (M2). Teeth with dental anomalies, periapical pathology, caries, restorations, severe attrition, or endodontic treatment were excluded. The selected scans were exported in DICOM format, anonymised by assigning random unique identification numbers, and imported into Mimics software (Materialise Mimics Research version 20.0, Materialise NV, Leuven, Belgium) for analysis.

### 2.3. Pulp Volume Segmentation

**Figure 1:**
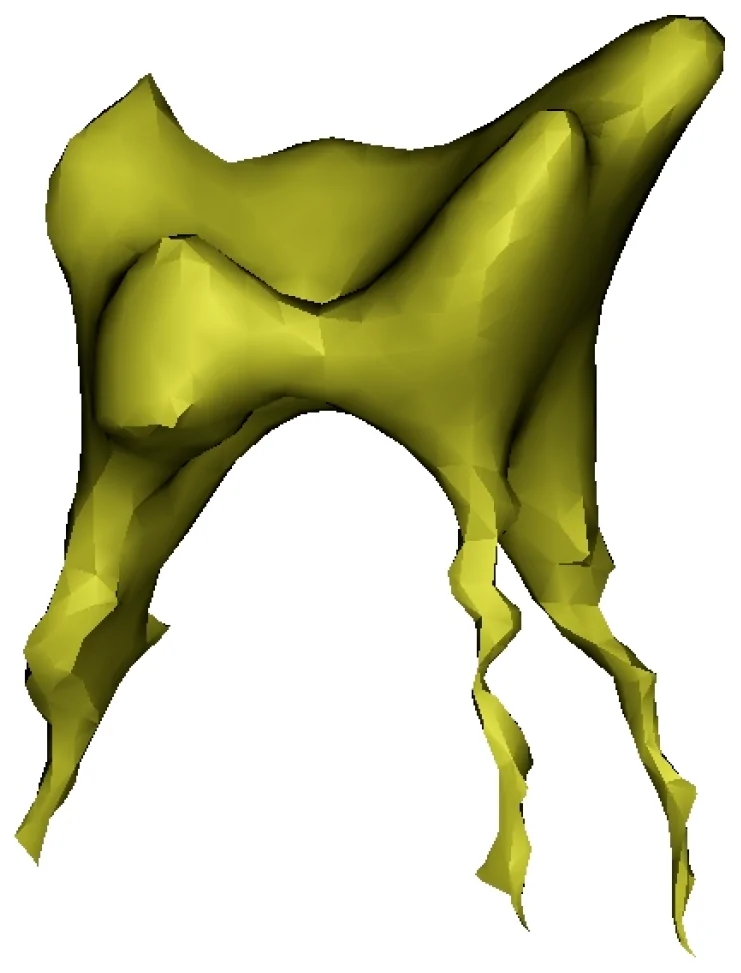
Three-dimensional smoothed surface reconstruction of a segmented mandibular molar pulp cavity derived from CBCT data.

All images were analysed by an oral and maxillofacial radiologist. To ensure optimal visualisation, the magnification, contrast, sharpness, and brightness settings were adjusted individually for each tooth. For each patient, tooth selection followed a hierarchical protocol: if both the left and right quadrants were eligible, the side demonstrating superior diagnostic image quality (sharpest pulpal contrast, minimal artefact exposure) was selected. In cases where both M1 and M2 were present and radiographically sound on the chosen side, selection was determined based on image quality; if quality was equivalent, M1 was prioritised unless excluded by exclusion criteria. For each tooth, the pulp outlines were verified slice-by-slice using the editing tool to ensure accurate calculation of pulp volume. The slice demonstrating the narrowest part of the pulp at the root apex was selected as the inferior limit of the measurement. Following the thresholding process, the segmented area was saved as a mask. A 3D smoothed surface model of the reconstructed pulp cavity was generated for qualitative visualisation (Figure 1). The pulp volume was calculated by multiplying the voxel volume by the number of voxels within the 3D model.

### 2.4. Statistical Analysis

Statistical analyses were conducted using IBM SPSS Statistics for Windows, Version 23.0 (IBM Corp., Armonk, NY, USA) and R statistical software, Version 4.3.1 (R Foundation for Statistical Computing, Vienna, Austria) [16]. Descriptive statistics, normality testing, between-group comparisons, correlation analyses, and Ordinary Least Squares (OLS) regression as a sensitivity analysis with bootstrapping were performed in SPSS. This version was retained for continuity with the analytical protocol used at the time of the original archival analysis. Huber M-estimator robust regression and quantile regression analyses were performed in R using the MASS package [17] and the quantreg package [18], respectively. Cross-validation was conducted using the caret package [19].

Descriptive statistics for quantitative variables were expressed as mean ± standard deviation (SD). Data normality was assessed using the Shapiro–Wilk test. Between-group comparisons were performed using the Mann–Whitney *U* test for non-normally distributed data and the independent samples *t*-test for normally distributed data. Due to the non-normal distribution of the pulp volume data, Spearman’s rank correlation analysis was employed to evaluate the strength and direction of the relationship between pulp volume and chronological age. Fisher’s *r*-to-*z* transformation was applied to compare correlation coefficients between sexes to identify potential sexual dimorphism in pulp reduction patterns. To verify that the two molar-type subgroups were age-comparable, the chronological age distributions of the M1 and M2 subgroups were compared using the Mann–Whitney U test, as both subgroups exhibited non-normal age distributions (Shapiro–Wilk test, both *p* < 0.001).

Prior to regression modelling, comprehensive diagnostics were performed for each molar subgroup to determine the most appropriate analytical approach. Residual normality was assessed using the Shapiro–Wilk test, homoscedasticity using the Breusch–Pagan test, and influential observations were identified using Cook’s distance (Cook’s *D*), with a threshold of 4/*n*. Residual plots and Q–Q plots were additionally inspected visually. Diagnostic results indicated that the assumptions underlying OLS regression were violated in both subgroups: the first molar group exhibited non-normal residuals (Shapiro–Wilk test, *p* = 0.007) with positive skewness (skewness = 1.375), while the second molar group demonstrated significant heteroscedasticity (Breusch–Pagan test, *p* = 0.011) and contained two high-leverage observations (Cook’s *D* = 0.773 and 0.261) with disproportionate influence on the regression slope.

Given these violations, Huber *M*-estimator robust regression (Huber’s *T* norm, tuning constant = 1.345) was selected as the primary analytical method for both subgroups, as it provides consistent and efficient parameter estimates under conditions of residual non-normality, heteroscedasticity, and the presence of influential observations [20,21]. OLS regression with bootstrapping (1000 resamples, BCa method) was retained as a sensitivity analysis to permit comparison with existing literature. Parametric model significance (F-tests and corresponding exact parametric *p*-values) and non-parametric BCa bootstrapped *p*-values were evaluated and reported independently to distinguish theoretical parametric fit from resampled inference. Quantile regression (τ = 0.25, 0.50, 0.75) was additionally performed as an exploratory analysis to characterise the relationship between pulp volume and age across the conditional distribution of chronological age [22].

Intra-observer reliability was evaluated by reanalysing a randomly selected subset of 15 teeth (25% of the total sample) after a two-week interval. The Intraclass Correlation Coefficient (ICC) was calculated using a two-way mixed-effects model with absolute agreement. The intra-observer ICC was 0.814 (95% CI: 0.756–0.913), indicating good repeatability based on the criteria of Koo and Li [23]. The threshold for statistical significance was established at *p* < 0.05.

As this study employed a retrospective design utilising archival CBCT data, prospective a priori power calculation was not feasible prior to data collection. Instead, a sensitivity analysis was conducted using G*Power (version 3.1.9.7; Heinrich Heine University Düsseldorf, Düsseldorf, Germany) to determine the minimum detectable effect size given the available sample. For simple linear regression with one predictor (α = 0.05, statistical power = 0.80), the overall sample (*n* = 60) is sufficient to detect an effect size of *f*^2^ ≥ 0.13, corresponding to an approximately moderate association. However, sex-stratified subgroup analyses were conducted with considerably smaller samples (M1: females, *n* = 18; males, *n* = 14; M2: females, *n* = 14; males, *n* = 14), and these results should be interpreted with caution given the reduced statistical power. In particular, the absence of a statistically significant correlation in the male first molar subgroup (*n* = 14; ρ = −0.339, *p* = 0.235) should be interpreted cautiously, as this sample size yields insufficient power to detect weak-to-moderate effect sizes and does not constitute evidence for the absence of a true association.

## 3. Results

The study cohort comprised 60 individuals, with a sex distribution of 32 females (53.33%) and 28 males (46.67%). The mean age of the overall sample was 24.32 ± 6.73 years (range: 18–48 years). A total of 60 mandibular molars were analysed, including 32 first molars (53.33%) and 28 second molars (46.67%). Descriptive statistics are summarised in Table 1. Female individuals demonstrated a mean pulp volume of 67.58 ± 18.94 mm^3^ (range: 31.7–107.0 mm^3^) and a mean age of 23.72 ± 6.53 years (range: 18–48 years). Male individuals exhibited a mean pulp volume of 58.39 ± 20.10 mm^3^ (range: 26.7–101.0 mm^3^) and a mean age of 25.00 ± 7.01 years (range: 18–46 years). No statistically significant differences were observed between sexes for either pulp volume or age (both *p* > 0.05). The chronological age distributions of the M1 and M2 subgroups were statistically comparable (M1: 24.78 ± 6.74 years, median 23.5 years, range 18–48 years; M2: 23.79 ± 6.80 years, median 21.0 years, range 18–46 years; Mann–Whitney U = 500.5, *p* = 0.439), indicating that the two tooth-type subgroups did not differ significantly with respect to the age of the individuals from whom measurements were obtained.

### 3.1. First Molar Group

In the first molar group, a statistically significant negative correlation was found between pulp volume and age in females (Spearman’s ρ = −0.669, *p* = 0.002; *n* = 18). In male individuals, a negative correlation was observed in the same direction, although it did not reach statistical significance (ρ = −0.339, *p* = 0.235; *n* = 14). When the sex-specific correlation coefficients were compared using the Fisher’s *r*-to-*z* transformation, no statistically significant difference was detected between the two sexes (*z* = 1.146, *p* = 0.252), indicating the absence of detectable sexual dimorphism in the volume–age relationship within this subgroup, though the limited male sample size warrants caution in this interpretation. For all first molars combined, the results revealed a statistically significant negative correlation (Spearman’s ρ = −0.554, *p* = 0.001).

**Figure 2:**
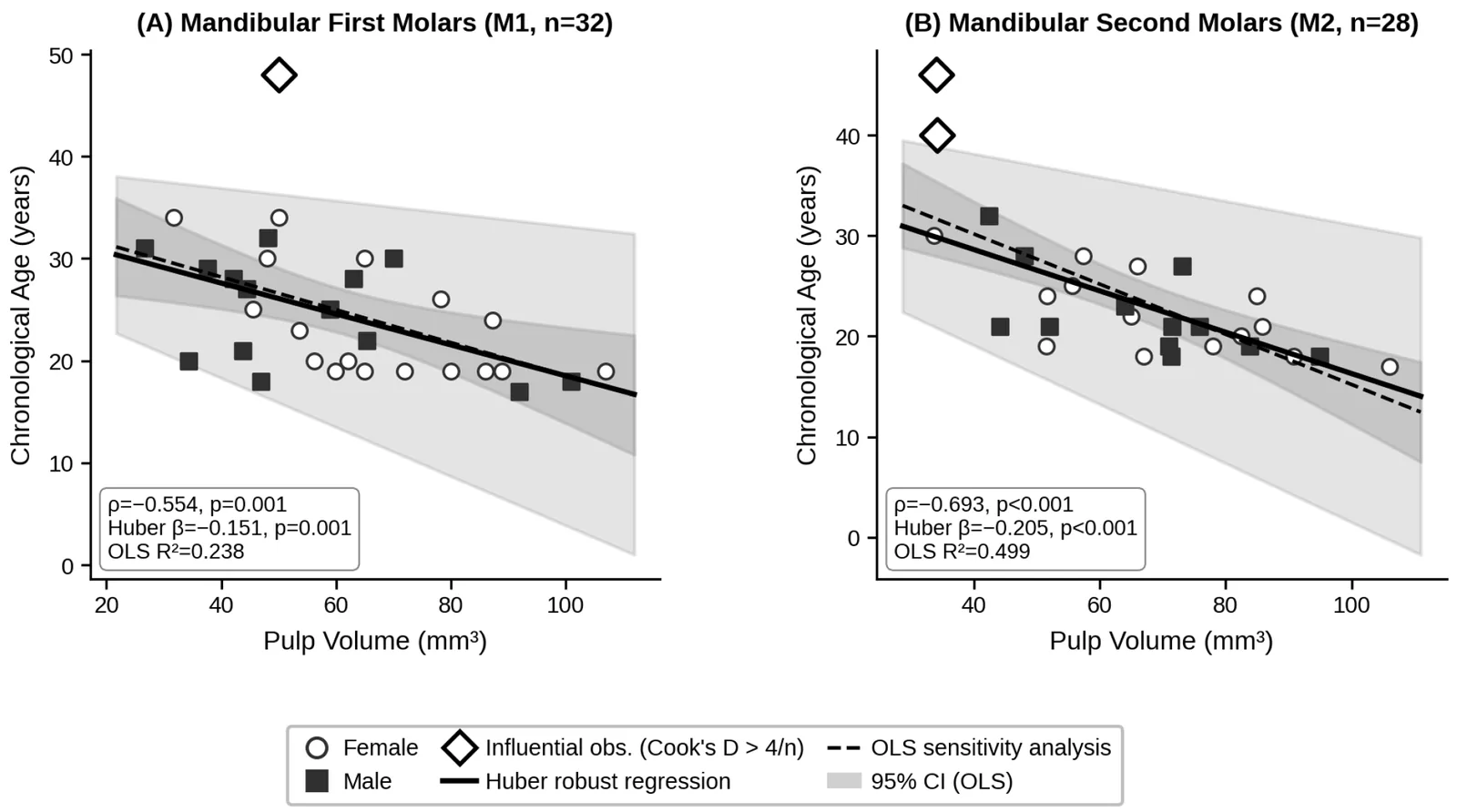
Scatter plots with Huber M-estimator robust and OLS sensitivity regression lines for mandibular first molars (**A**) and second molars (**B**). Circles: female; squares: male; diamonds: influential observations exceeding Cook’s distance threshold (4/*n*). Solid line: Huber robust regression (primary model); dashed line: OLS sensitivity analysis. Shaded bands: 95% confidence intervals. ρ: Spearman correlation coefficient; β: Huber slope estimate.

**Figure 3:**
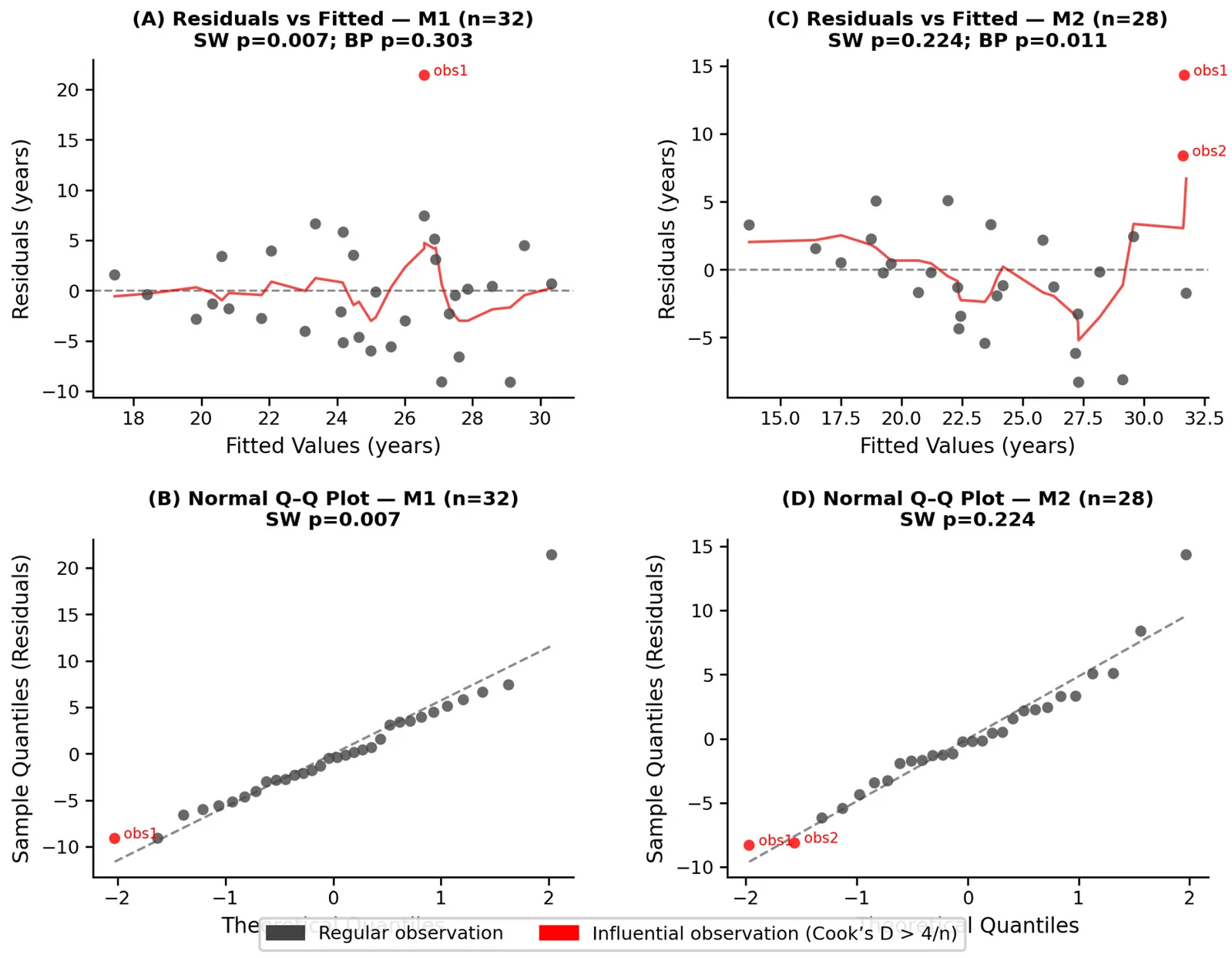
Residual diagnostic plots for the first molar (**A**,**B**) and second molar (**C**,**D**) OLS regression models. (**A**,**C**) Residuals versus fitted values with locally smoothed trend line (red); the horizontal dashed line indicates zero residual. (**B**,**D**) Normal Q–Q plots; the dashed line represents the theoretical normal reference line. Red circles indicate influential observations(Cook’s *D* > 4/*n*). Shapiro–Wilk (SW) and Breusch–Pagan (BP) *p*-values are shown in panel titles.

**Figure 4:**
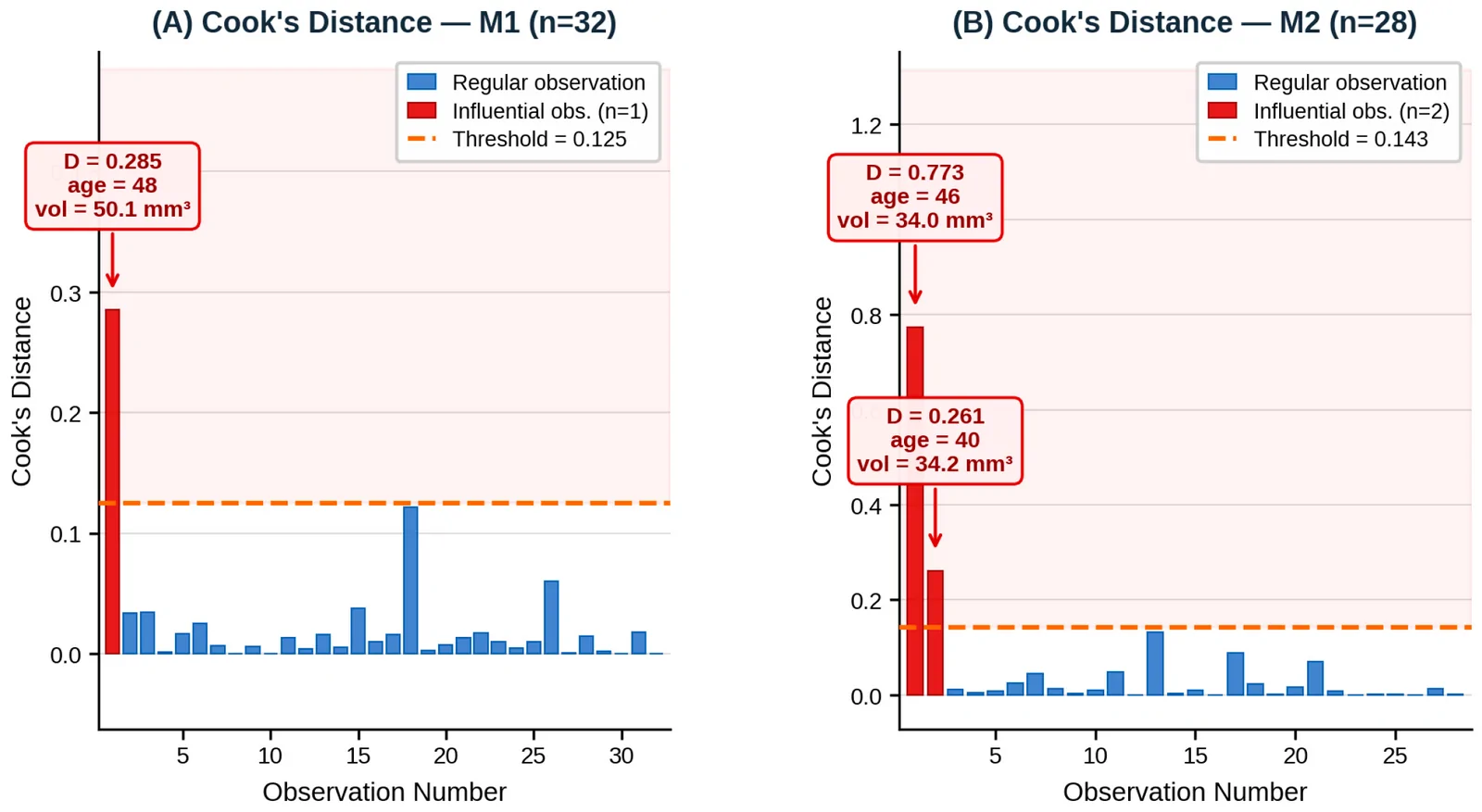
Cook’s distance for each observation in the first molar (**A**) and second molar (**B**) regression models. Red dashed line indicates the influence threshold (4/*n*). Bars exceeding the threshold (red) are labelled with Cook’s *D* value and the corresponding age and pulp volume of the observation.

The volume–age relationship in the first molar group, together with the fitted robust and OLS regression lines, is presented in Figure 2A. Regression diagnostics for the first molar group confirmed a violation of the residual normality assumption (Shapiro–Wilk test, *p* = 0.007; skewness = 1.375) (Figure 3A,B) and identified one influential observation (Cook’s *D* = 0.285; sex = female, volume = 50.1 mm^3^, age = 48 years), which exceeded the threshold of 4/*n* = 0.125 (Figure 4A). Accordingly, Huber *M*-estimator robust regression was applied as the primary model. Pulp volume was identified as a statistically significant negative predictor of chronological age (*B* = −0.151, *p* = 0.001; 95% CI: [−0.240, −0.062]). The robust regression equation was:

Age = 33.646 − 0.151 × Volume

As a sensitivity analysis, OLS regression with BCa bootstrapping (1000 resamples) yielded the parametric equation Age = 34.623 − 0.161 × Volume (*R*^2^ = 0.238, adjusted *R*^2^ = 0.213; SEE = ±5.978 years), with a slope estimate closely comparable to the robust estimate (−0.161 versus −0.151, approximately 7% larger in magnitude) and an intercept differing by less than one year (34.623 versus 33.646). The parametric OLS model was statistically significant (*F*(1,30) = 9.391, *p* = 0.005). The BCa 95% CI [−0.242, −0.080] for the slope did not include zero, supporting the statistical significance of the association under bootstrapped inference. The convergence of findings across both methods supports the robustness of the negative relationship between pulp volume and chronological age in the first molar group.

### 3.2. Second Molar Group

In the second molar group, statistically significant negative correlations were observed in both females (Spearman’s ρ = −0.615, *p* = 0.019; *n* = 14) and males (ρ = −0.746, *p* = 0.002; *n* = 14). When the correlation coefficients were compared using the Fisher’s *r*-to-*z* transformation, no statistically significant difference was detected between the two sexes (*z* = −0.580, *p* = 0.562), suggesting no statistically significant evidence of sexual dimorphism in the volume–age relationship within the second molar group. For all second molars combined, a strong and statistically significant negative correlation was found (Spearman’s ρ = −0.693, *p* < 0.001).

Regression diagnostics for the second molar group revealed significant heteroscedasticity (Breusch–Pagan *p* = 0.011) (Figure 3C,D) and identified two high-leverage observations (Cook’s *D* = 0.773 and 0.261; both: sex = male, volume = 34.0 and 34.2 mm^3^, ages = 46 and 40 years) (Figure 4B). The observation with Cook’s *D* = 0.773 substantially exceeded the threshold of 4/*n* = 0.143, indicating a disproportionate influence on the OLS regression slope. Huber *M*-estimator robust regression was therefore applied as the primary model. Pulp volume remained a statistically significant negative predictor of chronological age (*B* = −0.205, *p* < 0.001; 95% CI: [−0.293, −0.118]). The robust regression equation was:

Age = 36.833 − 0.205 × Volume

As a sensitivity analysis, OLS regression with BCa bootstrapping yielded a notably inflated slope estimate, with the parametric equation Age = 40.144 − 0.249 × Volume (*R*^2^ = 0.499, adjusted *R*^2^ = 0.479; SEE = ±4.908 years), approximately 21% larger in magnitude than the robust estimate—a discrepancy attributable to the leverage exerted by the two influential observations. The parametric OLS model was statistically significant (*F* (1,26) = 25.847, *p* < 0.001). The bootstrapping procedure yielded a BCa 95% confidence interval of [−0.379, −0.132] for the volume coefficient, confirming the significant negative relationship while revealing a wider range of uncertainty compared to standard parametric estimates. The robust regression estimate (*B* = −0.205) is considered the more reliable and conservative characterisation of the volume–age relationship in this subgroup (Figure 2B).

### 3.3. Cross-Validation and Quantile Regression

Cross-validated predictive performance (5-fold cross-validation) showed that the robust and OLS models performed comparably, with a modest advantage for the Huber model in mean absolute error. For the first molar group, the Huber model yielded a mean absolute error (MAE) of 5.20 years and a root mean square error (RMSE) of 6.85 years, marginally outperforming the OLS model (MAE = 5.25 years; RMSE = 6.90 years). For the second molar group, the Huber model achieved a lower MAE (4.30 vs. 4.50 years) but a comparable RMSE (6.21 vs. 6.20 years) relative to OLS. These cross-validated errors, which exceed the corresponding in-sample standard errors of estimate, further underscore the limited individual-level predictive precision of the models.

Exploratory quantile regression indicated that the negative association between pulp volume and age was statistically significant at the median and upper quantiles in both molar groups (M1: *Q* = 0.50, *B* = −0.192, *p* = 0.012; *Q* = 0.75, *B* = −0.191, *p* = 0.008; M2: *Q* = 0.50, *B* = −0.197, *p* = 0.002; *Q* = 0.75, *B* = −0.254, *p* = 0.005), but the association did not reach significance at *Q* = 0.25 in either group (M1: *p* = 0.467; M2: *p* = 0.239), suggesting the volume–age relationship was more pronounced at higher age quantiles within this sample. Findings from all analyses are summarised in Table 2.

## 4. Discussion

The progressive reduction in dental pulp volume attributable to continuous secondary dentine deposition represents a well-established biological marker for age-related dental changes. The present study found a statistically significant negative correlation between chronological age and pulp volume in both mandibular first and second molars. In first molars, Spearman correlation analysis revealed a moderate negative correlation (ρ = −0.554, *p* = 0.001), with the OLS sensitivity model accounting for approximately 23.8% of the variance in chronological age (*R*^2^ = 0.238). In second molars, a stronger negative correlation was observed (ρ = −0.693, *p* < 0.001), with pulp volume explaining 49.9% of the variance in age (*R*^2^ = 0.499). A fundamental interpretive constraint of this study, however, is the pronounced skew of the sample towards young adulthood (mean age: 24.32 ± 6.73 years), with only limited representation of participants aged over 35 years. The regression models were predominantly fitted to data points representing individuals aged 18–35 years and cannot be reliably extrapolated to individuals aged over 40 years—the very cohort in which volumetric age estimation would carry the greatest forensic utility.

These findings suggest that although the association between pulp volume and chronological age is statistically significant in both molar groups, the predictive strength is considerably greater for second molars than for first molars. Nevertheless, the moderate coefficients of determination indicate that pulp volume alone is not a sufficiently strong predictor of chronological age, and that additional variables must be considered to improve estimation accuracy.

In the present study, absolute pulp volume was utilised as the primary predictor rather than the pulp-to-tooth volume ratio, which is frequently cited in the literature. While volume ratios can compensate for individual variations in tooth size, recent evidence suggests that absolute volumetric measurements in multi-rooted teeth like molars can provide high-fidelity data when precisely segmented [24]. Furthermore, as a preliminary investigation, our aim was to establish the direct baseline relationship between secondary dentine-related pulp reduction and age prior to the incorporation of pulp-to-tooth volume ratios. Future studies should utilise tooth and pulp volume index to improve predictive accuracy.

The superior predictive performance of the second molar (*R*^2^ = 0.499) compared to the first molar (*R*^2^ = 0.238) is likely multifactorial and cannot be fully explained by the present dataset. In this context, it is worth noting that occlusal contact distribution may differ between molar types: González Sequeros et al. [25] reported that mandibular second molars demonstrated higher mean occlusal contact numbers than first molars during maximum intercuspation, suggesting potentially greater functional involvement of this tooth type. Whether such differential contact patterns translate into meaningful differences in dentine–pulp complex stimulation and secondary dentine deposition rates remains inconclusive, as the cited evidence reflects contact number rather than direct force measurement, and the present study did not assess attrition, parafunctional habits, or masticatory force distribution. An additional contributing factor may be the differential eruption chronology of the two molar types. Mandibular second molars erupt approximately two to three years later than first molars; consequently, even at an identical chronological age, second molars have been exposed to functional loading for a shorter duration, potentially resulting in a more linear and less individually variable secondary dentine deposition trajectory at the time of examination. In the present cohort, the age distributions of the M1 and M2 subgroups were statistically comparable (M1: 24.78 ± 6.74 years; M2: 23.79 ± 6.80 years; Mann–Whitney U = 500.5, *p* = 0.439), confirming that the observed difference in predictive performance is not attributable to a sampling age imbalance between tooth-type subgroups, and further supporting the interpretation that the superior *R*^2^ of the second molar reflects an intrinsic biological characteristic of that tooth type rather than a cohort artefact. The observed difference in predictive performance between molar types therefore remains a hypothesis-generating finding that requires prospective investigation with controlled functional assessments before any mechanistic conclusion can be drawn.

Kazmi et al. [26] reported an *R*^2^ of 0.330 for age estimation based on total pulp volume in canine teeth. Although direct comparison is not appropriate given the morphological and functional differences between canines and molars, the broadly comparable values of explained variance suggest that pulp volumetry retains moderate predictive utility regardless of tooth type. Nevertheless, a substantial portion of age-related variance in pulpal dimensions remains unaccounted for by volume alone, underscoring the need for multivariate approaches in future studies.

Compared to Song et al. [24], who reported an *R*^2^ of 0.662 for mandibular first molar pulp cavity volume using an automated U-Net deep learning segmentation approach at a higher voxel resolution (0.15 mm), the predictive accuracy of the first molar model in the present study was considerably lower (*R*^2^ = 0.238). While differences in segmentation methodology and voxel resolution may contribute to this discrepancy, the age distribution of the respective cohorts represents an equally plausible explanatory variable: a sample with broader and more balanced age representation would inherently yield stronger regression performance. Direct numerical comparison between studies with differing sample characteristics and methodologies carries limited interpretive validity. While fully automated deep-learning models offer speed, our semi-automatic thresholding approach with manual slice-by-slice verification allows for the identification of fine anatomical structures that automated models might overlook, thereby maintaining superior anatomical accuracy [27]. This expert-guided refinement process provides a reliable reference for verifying that only true pulpal boundaries are included in volumetric analysis.

In terms of sexual dimorphism, the present study found that the volume–age correlation reached statistical significance in both sexes for the second molar group (females: ρ = −0.615, *p* = 0.019; males: ρ = −0.746, *p* = 0.002), consistent with the findings of Song et al. [24], who reported no significant inter-sex differences in their automated molar analysis. However, in the first molar group, the correlation was statistically significant only in females (ρ = −0.669, *p* = 0.002), while the male subgroup did not reach significance (ρ = −0.339, *p* = 0.235). This sex-specific pattern in M1 warrants careful interpretation: given the small male sample size (*n* = 14) and the correspondingly limited statistical power, the non-significant result may reflect a Type II error rather than a genuine absence of association. Fisher’s *r*-to-*z* transformation confirmed no statistically significant difference between sex-specific coefficients in either molar group, indicating that the underlying biological relationship does not differ meaningfully between sexes. In contrast, Kazmi et al. [26] identified sex as a critical predictor for optimising diagnostic accuracy, though this finding may partly reflect sample size advantages in their cohort.

Quantile regression analyses revealed statistically significant associations at both the median (Q = 0.50) and upper quantile (Q = 0.75) in both molar groups, while Q = 0.25 did not reach significance. This pattern suggests that the volume–age relationship is detectable across a broad range of the age distribution, with the association strengthening at higher age quantiles—consistent with the progressive and cumulative nature of secondary dentine deposition with advancing age.

A specific methodological tension warrants explicit acknowledgement. Huber M-estimation attains stability by assigning reduced weights to observations with large residuals, and in the present sample these observations were not randomly distributed with respect to age. The influential observation in the first molar group belonged to the oldest individual in that subgroup (48 years; Cook’s *D* = 0.285), and the two influential observations in the second molar group belonged to the oldest individual in that subgroup (46 years) together with a further individual aged 40 years—all at the upper extreme of an age distribution with a mean of 24.32 ± 6.73 years. The robust estimator therefore achieved its stability by down-weighting, by construction, precisely those observations carrying the greatest leverage over the older segment of the age range, which is the segment of principal forensic interest. The magnitude of this effect differed markedly between subgroups: in the first molar group, the robust and OLS slopes were nearly coincident (−0.151 versus −0.161, approximately 7%), indicating that the single influential observation exerted limited leverage, whereas in the second molar group the OLS slope was approximately 21% larger in magnitude than the robust slope (−0.249 versus −0.205), so that the choice of estimator materially altered the fitted relationship. The consequence is directional rather than neutral: a flatter slope combined with a lower intercept yields systematically younger predicted ages at small pulp volumes, that is, in precisely the individuals the model is least able to characterise. The quantile regression pattern described above does not resolve this concern; if anything, it sharpens it, since an association that strengthens towards the upper conditional quantiles implies that the observations most informative about the volume–age relationship lie in the age range that the robust estimator down-weights most heavily. The two analyses therefore point in partially opposing directions with respect to older individuals, and neither can be adjudicated within a sample containing so few participants above 35 years. The reported coefficients should accordingly be regarded as conservative characterisations of the association within a predominantly young adult sample, and the behaviour of the volume–age relationship beyond approximately 35 years remains empirically undetermined in this dataset.

The decision to evaluate the pulp in its entirety versus isolating the coronal chamber is an important methodological factor. While contemporary investigations by Helmy et al. [28] and Sukhadeve et al. [29] have suggested that isolated pulp chamber volumetry offers an expedient approach for rapid age estimation—demonstrating significant predictive accuracy in Egyptian (*R*^2^ = 0.668) and Indian (*R*^2^ = 0.531) cohorts, respectively—this localised assessment may fail to capture the full spectrum of biological dental ageing. As shown by Sue et al. [30], the decrease in pulp chamber size levels off in older age groups, whereas assessment of the entire pulp space accounts for progressive mineral deposition throughout both the crown and radicular anatomy. This comprehensive volumetric analysis provides a more biologically representative model of age-related dental ageing.

The observation by Sue et al. [30] that the reduction in pulp chamber dimensions levels off in older age groups has a direct implication for the functional form adopted here. The models fitted in the present study, like most published volumetric age estimation models, assume a linear decrease in pulp volume with chronological age, and this assumption was not formally tested: no polynomial or transformed specification was fitted, and no formal test of curvature was performed. If secondary dentine apposition decelerates with advancing age, a linear specification would misrepresent the relationship at the upper end of the age range. Within the present cohort, which was heavily skewed towards individuals under 35 years, the available age range is too narrow and its upper tail too sparse for departures from linearity to be reliably detectable. The linear specification should therefore be regarded as a locally applicable approximation rather than as an empirically supported functional form, and logarithmic and higher-order specifications should be examined formally in age-balanced cohorts spanning a wider age range.

### Limitations

The present study is subject to several limitations. A primary concern involves the selection of first and second molars; due to their early eruption and prolonged exposure to masticatory forces, these teeth are inherently more susceptible to carious lesions and restorative interventions, precluding the application of our methodology to teeth presenting with decay or pathological pulp reduction.

Furthermore, the age distribution presents a significant constraint. Although the declared age range spanned 18–48 years, the mean age was 24.32 ± 6.73 years, reflecting a pronounced skewness towards young adulthood. The regression equations derived here cannot be reliably extrapolated to individuals aged over 40 years, and the significant correlations observed may partly reflect natural variance in pulp volume among young adults due to inter-individual developmental differences, rather than solely chronological secondary dentine deposition. Future studies must recruit participants across a balanced age distribution spanning at least 18–70 years.

Additionally, the present study assessed absolute pulp volume rather than pulp-to-tooth volume ratios, intra-observer reliability was based on a single observer without inter-observer validation, and the universal applicability of these predictive models across diverse ethnic and geographical populations remains to be prospectively validated. In addition, pulp dimensions are influenced by factors beyond chronological age—including inter-individual variation and potential systemic or functional influences on secondary dentine deposition—that were not measured in this study and may partly confound the observed associations. Moreover, while our semi-automatic workflow ensures high anatomical fidelity through expert refinement, it remains more time-demanding than fully automated deep-learning models.

A further methodological consideration concerns the segmentation platform. The present pulp volumes were obtained by semi-automatic thresholding in a single software package (Mimics), and absolute volumetric outputs may be influenced by both the software and the acquisition protocol. Comparative validation studies provide a nuanced picture: established segmentation packages have frequently yielded broadly comparable volumes, with no significant differences reported among Mimics, OnDemand, and InVesalius for jaw-lesion volumes [31], between ITK-SNAP and a validated commercial package for pharyngeal volumes [32], or between ITK-SNAP and 3D Doctor for maxillary-defect volumes [33]. Meaningful discrepancies, however, can arise from other sources: segmentation-based volumes have been shown to diverge from formula-based estimates and to underestimate a physical gold standard [31], acquisition settings such as kVp significantly affected volumetric accuracy in vitro [33], and a specific commercial tool exhibited systematic bias relative to the gold standard and to well-validated packages such as ITK-SNAP and 3D Slicer [34]. Collectively, these findings suggest that the absolute pulp volumes reported here are unlikely to be arbitrary artefacts of the chosen software, yet they are not guaranteed to be fully transferable across platforms and acquisition protocols. The derived regression coefficients should therefore be regarded as specific to the present segmentation workflow and acquisition parameters, and cross-software and cross-protocol validation would be required before any external application.

The analytical framework incorporated stratification by molar type and sex along with exploratory quantile regressions, inherently increasing susceptibility to Type I error accumulation. Because the analytical strategy was hierarchically structured around two principal questions—specifically, evaluating the association between pulp volume and chronological age in first and second molars—no formal adjustment for multiplicity was implemented. Sex-stratified correlations and quantile regressions were designated as exploratory, hypothesis-generating procedures. To maintain complete reporting transparency, 21 inferential tests are documented. Under a conservative Bonferroni threshold (α = 0.05/21 = 0.002), the primary associations in both molar categories remained statistically significant across Spearman rank correlations and Huber robust regressions (all *p* ≤ 0.001). Conversely, the sex-stratified correlation in female second molars (*p* = 0.019), the parametric ordinary least squares *F*-test for first molars (*p* = 0.005), and several quantile-specific estimates did not survive adjustment. These exploratory outcomes should therefore be regarded as descriptive patterns specific to the evaluated sample rather than confirmatory associations, underscoring the need for formal familywise error rate or false discovery rate control in future adequately powered multivariate investigations.

## 5. Conclusions

This preliminary study demonstrates a statistically significant negative correlation between CBCT-derived pulp volume and chronological age in mandibular first and second molars in a Turkish population sample. Huber *M*-estimator robust regression—selected following confirmation of OLS assumption violations through comprehensive diagnostic testing—confirmed pulp volume as a significant predictor in both groups, with the mandibular second molar yielding a stronger association (β = −0.205, *p* < 0.001; OLS *R*^2^ = 0.499, SEE = ±4.908 years) than the first molar (β = −0.151, *p* = 0.001; OLS *R*^2^ = 0.238, SEE = ±5.978 years). However, these findings must be interpreted within the substantial constraints of the study: the sample was predominantly composed of young adults, the explained variance remained below 50% even in the better-performing model, and standard errors of estimate exceeding 4.9 years preclude individual-level forensic age determination in their current form. The contribution of this study lies in providing preliminary baseline data on the pulp volume–age relationship in Turkish mandibular molars and in identifying the methodological refinements—including age-balanced sampling, pulp-to-tooth volume ratios, multivariate modelling, inter-observer reliability validation, and prospective design—necessary for the development of forensically applicable age estimation tools.

## Figures and Tables

**Table 1:** Descriptive statistics of the study sample stratified by sex and molar type.

Parameter	Female (*n* = 32)	Male (*n* = 28)	*p*-Value
Age (years)			
Mean ± SD	23.72 ± 6.53	25.00 ± 7.01	>0.05
Median (IQR)	21.0 (19–27)	22.5 (19–30)	
Range	18–48	18–46	
Pulp Volume (mm^3^)			
Mean ± SD	67.58 ± 18.94	58.39 ± 20.10	>0.05
Median (IQR)	66.5 (52–80)	57.7 (44–71)	
Range	31.7–107.0	26.7–101.0	
Molar Type—*n* (%)			
First molar (M1)	18 (56.25%)	14 (50.00%)	
Second molar (M2)	14 (43.75%)	14 (50.00%)	
M1 (*n* = 32)	Female (*n* = 18)	Male (*n* = 14)	
Age—Mean ± SD (years)	24.83 ± 7.85	24.71 ± 5.25	
Volume—Mean ± SD (mm^3^)	65.93 ± 19.06	55.32 ± 21.40	
M2 (*n* = 28)	Female (*n* = 14)	Male (*n* = 14)	
Age—Mean ± SD (years)	22.29 ± 4.12	25.29 ± 8.62	
Volume—Mean ± SD (mm^3^)	69.71 ± 19.29	61.46 ± 19.00	

IQR: Interquartile Range; SD: Standard Deviation.

**Table 2:** Primary (Huber robust) and sensitivity (OLS bootstrapped) regression results for mandibular molar pulp volume as a predictor of chronological age.

Parameter	First Molars (*n* = 32)	Second Molars (*n* = 28)
Spearman Correlation (ρ)		
Overall (ρ)	−0.554 **	−0.693 **
Female (ρ)	−0.669 **	−0.615 *
Male (ρ)	−0.339 (*p* = 0.235)	−0.746 **
Fisher’s *r*-to-*z* (*z*; *p*)	1.146; 0.252	−0.580; 0.562
Primary: Huber Robust Regression		
Intercept	33.646	36.833
β (volume)	−0.151	−0.205
95% CI for β	[−0.240, −0.062]	[−0.293, −0.118]
*p*-value	0.001	<0.001
Sensitivity: OLS + BCa Bootstrap		
Intercept	34.623	40.144
B (volume)	−0.161	−0.249
95% BCa CI for B	[−0.242, −0.080]	[−0.379, −0.132]
Parametric Model F-test (*p*-value)	*F*(1, 30) = 9.391, *p* = 0.005	*F*(1, 26) = 25.847, *p* < 0.001
Bootstrap *p*-value	0.002	0.002
*R* ^2^	0.238	0.499
Adjusted *R*^2^	0.213	0.479
SEE (years)	±5.978	±4.908
Cross-validation (5-fold)		
Huber MAE (years)	5.20	4.30
OLS MAE (years)	5.25	4.50
Diagnostic Tests		
Residual normality (Shapiro–Wilk *p*)	0.007 (violated)	0.224 (met)
Homoscedasticity (Breusch–Pagan *p*)	0.303 (met)	0.011 (violated)
Cook’s *D* > 4/*n*	1 obs (*D* = 0.285)	2 obs (*D* = 0.773, 0.261)

* *p* < 0.05, ** *p* < 0.01. SEE: Standard Error of Estimate; BCa: Bias-Corrected and Accelerated; MAE: Mean Absolute Error; CI: Confidence Interval. Robust regression: Huber M-estimator (tuning constant = 1.345). OLS sensitivity analysis based on 1000 BCa bootstrap resamples. Parametric OLS regression *p*-values reflect the *F*-test model significance (*p* = 0.005 for M1; *p* < 0.001 for M2) and are reported independently of the non-parametric BCa bootstrap resampling *p*-values (*p* = 0.002 for both models).

## Data Availability

The data presented in this study are available on request from the corresponding author. The data are not publicly available due to privacy and ethical restrictions.
